# Incidental Arrhythmias During Atrial Fibrillation Screening With Repeat 7‐Day Holter ECGs in a Hospital‐Based Patient Population

**DOI:** 10.1161/JAHA.123.032223

**Published:** 2024-02-13

**Authors:** Eleni Goulouti, Anna Lam, Nikolas Nozica, Elena Elchinova, Chrisoula Dernektsi, Felix Neugebauer, Mattia Branca, Helge Servatius, Fabian Noti, Andreas Haeberlin, Gregor Thalmann, Nikola Asenov Kozhuharov, Antonio Madaffari, Hildegard Tanner, Tobias Reichlin, Laurent Roten

**Affiliations:** ^1^ Department of Cardiology Inselspital, Bern University Hospital, University of Bern Bern Switzerland; ^2^ Clinical Trials Unit University of Bern Switzerland; ^3^ Sitem Center for Translational Medicine and Biomedical Entrepreneurship University of Bern Switzerland

**Keywords:** atrial fibrillation, atrioventricular block, Holter ECG, pacemaker, screening, sinus node dysfunction, supraventricular tachycardia, Arrhythmias

## Abstract

**Background:**

Screening for atrial fibrillation (AF) may reveal incidental arrhythmias of relevance. The aim of this study was to describe incidental arrhythmias detected during screening for AF in the STAR‐FIB (Predicting SilenT AtRial FIBrillation in Patients at High Thrombembolic Risk) cohort study.

**Methods and Results:**

In the STAR‐FIB cohort study, we screened hospitalized patients for AF with 3 repeat 7‐day Holter ECGs. We analyzed all Holter ECGs for the presence of the following incidental arrhythmias: (1) sinus node dysfunction, defined as sinus pause of ≥3 seconds’ duration; (2) second‐degree (including Wenckebach) or higher‐degree atrioventricular block (AVB); (3) sustained supraventricular tachycardia of ≥30 seconds’ duration; and (4) sustained ventricular tachycardia of ≥30 seconds’ duration. We furthermore report treatment decisions because of incidental arrhythmias. A total of 2077 Holter ECGs were performed in 794 patients (mean age, 74.7 years; 49% women), resulting in a mean cumulative duration of analyzable ECG signal of 414±136 hours/patient. We found incidental arrhythmias in 94 patients (11.8%). Among these were sinus node dysfunction in 14 patients (1.8%), AVB in 41 (5.2%), supraventricular tachycardia in 42 (5.3%), and ventricular tachycardia in 2 (0.3%). Second‐degree AVB was found in 23 patients (2.9%), 2:1 AVB in 10 (1.3%), and complete AVB in 8 (1%). Subsequently, 8 patients underwent pacemaker implantation, 1 for sinus node dysfunction (post‐AF conversion pause of 9 seconds) and 7 for advanced AVB. One patient had an implantable cardioverter‐defibrillator implanted for syncopal ventricular tachycardia.

**Conclusions:**

Incidental arrhythmias were frequently detected during screening for AF in the STAR‐FIB study and resulted in device therapy in 1.1% of our cohort patients.

**Nonstandard Abbreviations and Acronyms**
AVB: atrioventricular block
SND: sinus node dysfunction
SVT: supraventricular tachycardia

**Clinical Perspective**

**What Is New?**

- Screening for atrial fibrillation in a hospitalized patient population without known atrial fibrillation by repeat 7‐day Holter ECGs revealed incidental arrhythmias in 12% of patients.
- Sinus node dysfunction was observed in 1.8% of patients, second‐ or higher‐degree atrioventricular block in 5.3%, and sustained ventricular tachycardia in 0.3% and resulted in pacemaker or implantable cardioverter implantation in 1.1% of patients.

**What Are the Clinical Implications?**

- Incidental arrhythmias detected during screening for atrial fibrillation need to be taken into account before starting a screening program and often need evaluation and patient counseling by an experienced electrophysiologist to avoid overtreatment and unsettling of patients.

In our aging Western societies, the prevalence of atrial fibrillation (AF) is expanding rapidly, and so are associated morbidity and mortality.^1^ Early recognition and treatment of AF have gained increased interest. Numerous AF screening studies using various screening tools have been published in recent years.^2^, ^3^, ^4^ Although evidence on stroke prevention by AF screening remains ambiguous,^5^, ^6^ several ambitious randomized controlled studies are underway that will further investigate whether AF screening can reduce the incidence of stroke.^7^, ^8^, ^9^, ^10^

Screening for a disease may lead to the fortuitous detection of another disease. In the case of AF screening, incidental arrhythmias may be found that cannot be ignored and necessitate comprehensive evaluation and medical treatment. AF screening may also have adverse effects because of the screening tool used, like skin reaction or infection of an implanted cardiac monitor. All of this needs to be considered before a screening program is established, as it may influence the benefit of AF screening as well as cost estimates. However, most AF screening studies fail to report in detail incidental arrhythmias diagnosed during AF screening and the adverse effects of the screening tool.

In the prospective STAR‐FIB (Predicting SilenT AtRial FIBrillation in Patients at High Thrombembolic Risk) cohort study, we recruited hospitalized patients and screened them for AF with repeat Holter ECGs.^11^ The present study aims to report in detail incidental arrhythmias diagnosed during the 7‐day Holter ECGs of the STAR‐FIB study participants and subsequent treatment decisions.

## Methods

The STAR‐FIB study program comprises a hospital‐based, prospective cohort study and a hospital‐based, case‐control study. The cohort study aimed to determine the age‐ and the sex‐specific and overall prevalence of silent AF. The design and rationale of the STAR‐FIB study program and the results of the prospective cohort study have been reported elsewhere.^11^, ^12^ Patients were recruited from the Departments of Internal Medicine, Cardiology, and Ophthalmology of the University Hospital of Bern. In brief, we included 795 patients, aged 65 to 84 years, without AF in the prospective cohort study. More important, we stratified inclusion by age and sex and thereby included a similar number of men and women in 4 age groups (65–69, 70–74, 75–79, and 80–84 years). Patients with an implanted cardiac electronic device or with AF were excluded from participation in the STAR‐FIB study program. All patients had an ECG, signal‐averaged ECG, echocardiography, and three 7‐day Holter ECGs. Echocardiography included measurement of left atrial volume index, left ventricular myocardial mass index, and time interval from the beginning of the *P* wave on the surface ECG to the peak A' wave on the tissue‐Doppler imaging tracing of the lateral left atrial wall. The study complies with the Declaration of Helsinki and was approved by the locally appointed ethics committee of the Canton of Bern (KEK‐BE 257/14). All study participants provided written informed consent. The data that support the findings of this study are available from the corresponding author upon reasonable request.

### The 7‐Day Holter ECG

The study participants had three 7‐day Holter ECGs in 2‐month intervals. The first Holter ECG was recorded on hospital discharge, and the subsequent 2 Holter ECGs were recorded on an outpatient basis. If AF was diagnosed, subsequent Holter ECGs were canceled. We used the Lifecard CF Holter system (Spacelabs Healthcare, Issaquah, WA) and recorded 2 ECG channels during the entire 7 days. Complete recordings were analyzed for the presence of AF and other, incidental arrhythmias with the Pathfinder SL software (Spacelabs Healthcare) with additional manual confirmatory analysis. All patients had to complete a diary describing their activities and symptoms during the 7 days they wore the Holter ECG. They were explicitly asked to document whether they experienced any adverse effects of the Holter ECG.

### Incidental Arrhythmias

We classified incidental arrhythmias in the 7‐day Holter ECGs as follows: (1) sinus node dysfunction (SND), defined as sinus pause of ≥3 seconds’ duration; (2) second‐degree (including Wenckebach) or higher‐degree atrioventricular block (AVB); (3) sustained supraventricular tachycardia (SVT) of ≥30 seconds’ duration; and (4) sustained ventricular tachycardia (VT) of ≥30 seconds’ duration. Patients with clinically relevant, incidental arrhythmias were offered comprehensive evaluation and counseling by an experienced electrophysiologist on further treatment options. Indications for device implantation followed recommendations of current guidelines.

### Statistical Analysis

Continuous variables are expressed as means with SDs, and categorical variables are expressed as frequencies with percentages. Continuous variables were compared using the Mann‐Whitney *U* test or *t* test. Differences in proportions were tested with the Pearson χ^2^ test or the Fisher exact test, as appropriate. Multivariable analysis was performed to find potential predictors for incidental findings using a logistic regression with robust SEs. Variables were preselected for the multivariable analysis if they had a *P*<0.10 on the univariable comparison. Multiple imputation was applied to those variables with missing values, applying multiple imputation by chained equation on 50 data sets and using the complete characteristics as predictors for the imputed missing values. Backward variable selection was applied to the preselected model, with the selection criteria being a *P*<0.05. All statistical analyses were performed using Stata 17.0 (StataCorp, College Station, TX).

## Results

A total of 794 patients were enrolled (mean age, 74.7 years; 49% women). In total, we performed two thousand seventy‐seven 7‐day Holter ECGs (3 in 616 patients, 2 in 51 patients, and 1 in 127 patients). The mean±SD cumulative duration of an analyzable ECG signal recording in the 7‐day Holter ECGs was 414±136 hours per patient (156±28, 160±19, and 161±18 hours for the first, second, and third 7‐day Holter ECG, respectively). AF was newly diagnosed in 29 patients during a 7‐day Holter ECG (3.7%).^11^

**Figure 1:**
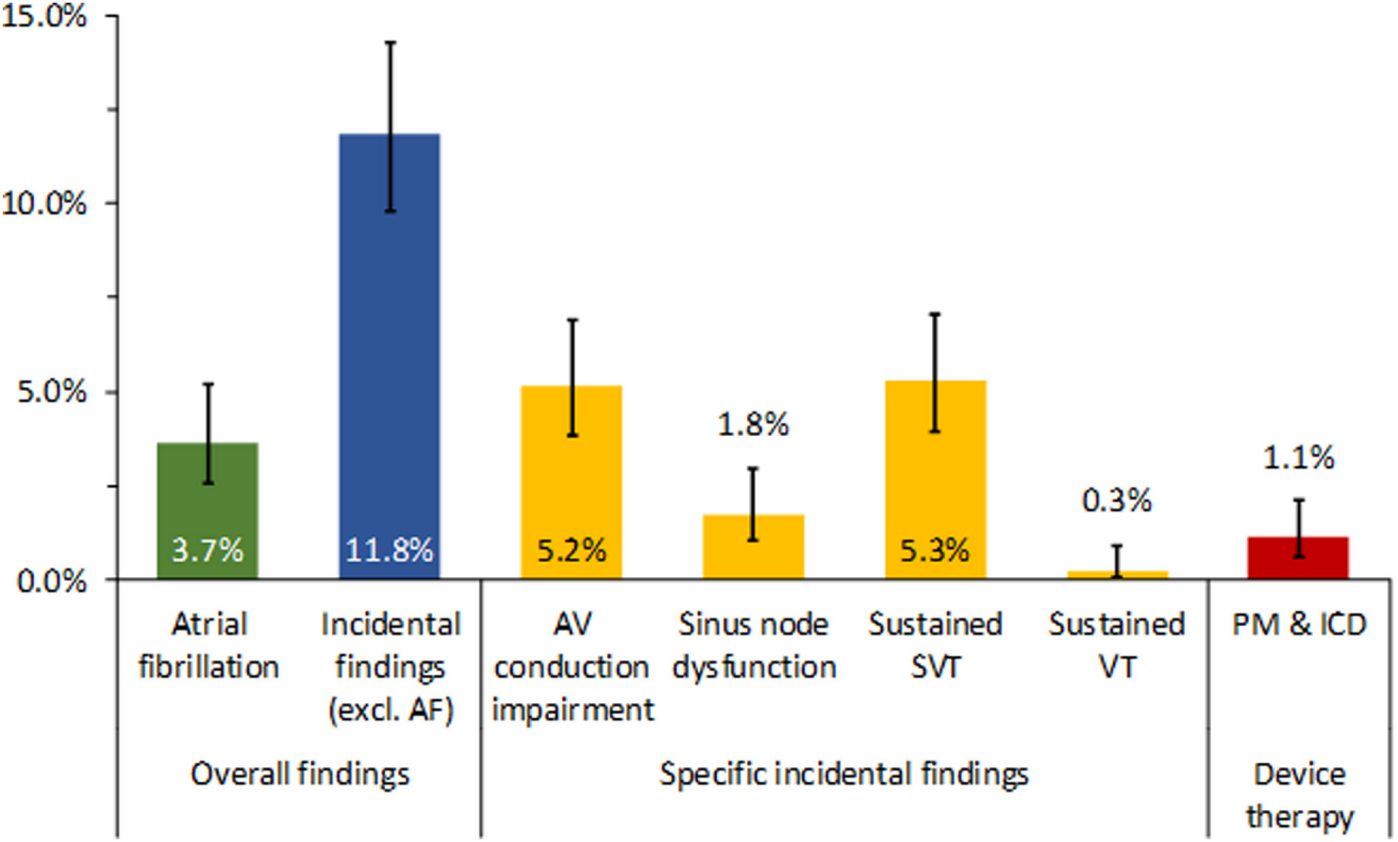
Incidence of incidental findings and consecutive device therapy during AF screening with Holter ECG. Bar graph showing the percentage (with 95% CIs) of patients with AF, incidental findings, different types of incidental findings, and device therapy. AF indicates atrial fibrillation; AV, atrioventricular; ICD, implantable cardioverter‐defibrillator; PM, pacemaker; SVT, supraventricular tachycardia; and VT, ventricular tachycardia.

We observed incidental arrhythmias in 94 patients (11.8%; Figure 1). Table 1 shows the characteristics of all patients and of patients with and without incidental arrhythmias. Patients with incidental arrhythmias were older, were more likely men, had longer PR interval and filtered *P‐*wave duration, had larger left ventricular myocardial mass index, left atrial volume index, and total atrial conduction time as assessed by tissue Doppler imaging (PA‐TDI), and had higher creatinine and brain natriuretic peptide levels (Table 1). In the multivariable analysis, PR interval and hs‐CRP (high‐sensitivity C‐reactive protein) remained significant predictors of incidental arrhythmias (Table 2).

**Table 1:** Patient Characteristics Overall, and in Patients With or Without Incidental Arrhythmias in a 7‐Day Holter ECG

Characteristic	All (N=794)	No incidental arrhythmias (N=700)	Incidental arrhythmias (N=94)	*P* value
Clinical characteristics				
Age, y	74.7±5.6	74.5±5.7	76.0±5.2	0.018
Sex, women	390 (49)	354 (51)	36 (38)	0.025
BMI, kg/m^2^	26.5±4.6	26.5±4.6	26.6±5.1	0.905
Palpitations in past 12 mo	186 (23)	157 (22)	29 (31)	0.070
Arterial hypertension	556 (70)	492 (70)	64 (68)	0.662
Diabetes	152 (19)	136 (19)	16 (17)	0.578
Dyslipidemia	418 (53)	365 (52)	53 (56)	0.439
Coronary artery disease	246 (31)	220 (31)	26 (28)	0.458
Peripheral artery disease	53 (7)	43 (7)	10 (11)	0.105
Congestive heart failure	18 (2)	15 (2)	3 (3)	0.538
Previous thrombotic event	121 (15)	105 (15)	16 (17)	0.572
Stroke	66 (8)	55 (8)	11 (12)	0.205
Transient ischemic attack	52 (7)	46 (7)	6 (6)	0.945
Peripheral embolism	9 (1)	9 (1)	…	0.609
β‐Blocker	285 (36)	250 (36)	35 (37)	0.773
Calcium channel blocker	6 (1)	5 (1)	1 (1)	0.534
Digoxin	1 (0)	1 (0)	…	1.000
ECG				
PR interval, ms	174±31	171±29	194±38	<0.001
QRS width, ms	94±20	94±20	95±18	0.737
QTc, ms	436±27	437±27	434±26	0.305
SAECG				
Filtered *P‐*wave duration, ms	141±17	140±16	146±17	0.002
Echocardiography				
LVEF, %	62±6	62±6	61±5	0.222
LVEDD, mm	47±7	47±7	49±7	0.063
LVMMI, g/m^2^	116±36	115±35	126±41	0.010
LAVI, mL/m^2^	29±10	29±9	32±11	0.018
PA‐TDI, ms	132±25	131±25	138±24	0.013
Laboratory analysis				
Creatinine, μmol/L	89±27	89±25	96±38	0.020
hs‐CRP, mg/L (sqrt)	1.48±0.89	1.46±0.86	1.65±1.09	0.077
BNP, pg/mL (ln)	3.87±0.90	3.84±0.90	4.10±0.90	0.012
hs‐TNT, μg/L (sqrt)	0.11±0.05	0.11±0.05	0.12±0.04	0.167

Data are shown as number (percentage) or mean±SD, as appropriate. BMI indicates body mass index; BNP, brain natriuretic peptide; hs‐CRP, high‐sensitivity C‐reactive protein; hs‐TNT, high‐sensitivity troponin T; LAVI, left atrial volume index; LVEDD, left ventricular end‐diastolic diameter; LVEF, left ventricular ejection fraction; LVMMI, left ventricular myocardial mass index; PA‐TDI, total atrial conduction time as assessed by tissue Doppler imaging; QTc, corrected QT interval; SAECG, signal‐averaged ECG; and sqrt, square root.

**Table 2:** Multivariable Analysis of Predictors of Incidental Arrhythmias

	Full model			Selected model		
Variable	Coefficient (95% CI)	aOR (95% CI)	*P* value	Coefficient (95% CI)	aOR (95% CI)	*P* value
Age, y	0.01 (−0.03 to 0.05)	1.01 (0.97 to 1.05)	0.638	…	…	…
Sex, women	−0.25 (−0.84 to 0.33)	0.78 (0.43 to 1.40)	0.398	…	…	…
Palpitations in past 12 mo	0.55 (0.03 to 1.08)	1.74 (1.03 to 2.94)	0.040	…	…	…
PR interval, ms	0.02 (0.01 to 0.03)	1.02 (1.01 to 1.03)	<0.001	0.02 (0.01 to 0.03)	1.02 (1.01 to 1.03)	<0.001
Filtered *P‐*wave duration, ms	−0.00 (−0.02 to 0.02)	1.00 (0.98 to 1.02)	0.986	…	…	…
LVEF, %	0.00 (−0.03 to 0.04)	1.00 (0.97 to 1.04)	0.777	…	…	…
LVEDD, mm	0.01 (−0.04 to 0.06)	1.01 (0.96 to 1.06)	0.616	…	…	…
LVMMI, g/m^2^	0.00 (−0.01 to 0.01)	1.00 (0.99 to 1.01)	0.677	…	…	…
LAVI, mL/m^2^	0.00 (−0.02 to 0.03)	1.00 (0.98 to 1.03)	0.783	…	…	…
PA‐TDI, ms	0.01 (−0.00 to 0.02)	1.01 (1.00 to 1.02)	0.249	…	…	…
Creatinine, μmol/L	0.00 (−0.00 to 0.01)	1.00 (1.00 to 1.01)	0.325	…	…	…
hs‐CRP, mg/L (sqrt)	0.06 (0.00 to 0.11)	1.06 (1.00 to 1.12)	0.036	0.26 (0.01 to 0.52)	1.30 (1.01 to 1.67)	0.042
BNP, pg/mL (ln)	0.00 (−0.00 to 0.00)	1.00 (1.00 to 1.00)	0.704	…	…	…

aOR indicates adjusted odds ratio; BNP, brain natriuretic peptide; hs‐CRP, high‐sensitivity C‐reactive protein; LAVI, left atrial volume index; LVEDD, left ventricular end‐diastolic diameter; LVEF, left ventricular ejection fraction; LVMMI, left ventricular myocardial mass index; PA‐TDI, total atrial conduction time as assessed by tissue Doppler imaging; and sqrt, square root.

### Atrioventricular Block

We observed an AVB in 41 patients (5.2%; Table 3, Figure 1). The most severe type of AVB found per patient was second‐degree AVB type Wenckebach in 23 patients (2.9%), 2:1 atrioventricular conduction in 10 patients (1.3%), and complete AVB in 8 patients (1%). Seven of the patients with complete AVB had a pacemaker implanted (Table S1). Three patients with complete AVB had a junctional escape rhythm without a pause, and 5 had pauses of ≥3 seconds. Age, PR interval, left ventricular end‐diastolic volume, left ventricular myocardial mass index, and PA‐TDI were predictors for incidental AVB in the univariable analysis (Table 3), but only the PR interval remained predictive in the multivariable analysis (Table 4). Manifestation of AVB mainly occurred in the first 7‐day Holter ECG, and incidence decreased during subsequent 7‐day Holter ECGs (Table 5).

**Table 3:** Patient Characteristics of Patients With Specific Incidental Findings in a 7‐Day Holter ECG

Characteristic	No sustained SVT (N=752)	Sustained SVT (N=42)	*P* value	No SND (N=780)	SND (N=14)	*P* value	No atrioventricular conduction impairment (N=753)	Atrioventricular conduction impairment (N=41)	*P* value
Clinical characteristics									
Age, y	74.6±5.7	75.8±4.9	0.193	74.7±5.6	76.1±5.9	0.364	74.6±5.6	76.7±5.4	0.019
Sex, women	373 (50)	17 (40)	0.250	386 (49)	4 (29)	0.121	375 (50)	15 (37)	0.099
BMI, kg/m^2^	26.5±4.7	25.9±3.3	0.416	26.5±4.6	28.3±6.8	0.160	26.5±4.6	26.7±5.8	0.787
Palpitations in past 12 mo	172 (23)	14 (33)	0.119	183 (23)	3 (21)	0.859	172 (23)	14 (34)	0.096
Arterial hypertension	526 (70)	30 (71)	0.838	543 (70)	13 (93)	0.060	531 (71)	25 (61)	0.194
Diabetes	146 (19)	6 (14)	0.411	148 (19)	4 (29)	0.366	146 (19)	6 (15)	0.451
Dyslipidemia	393 (52)	25 (60)	0.359	409 (52)	9 (64)	0.379	396 (53)	22 (54)	0.894
Coronary artery disease	238 (32)	8 (19)	0.086	239 (31)	7 (50)	0.121	232 (31)	14 (34)	0.653
Peripheral artery disease	657 (93)	37 (95)	0.623	684 (93)	10 (71)	0.002	660 (93)	34 (87)	0.153
Congestive heart failure	718 (98)	39 (95)	0.264	743 (98)	14 (100)	0.560	717 (98)	40 (98)	0.959
Previous thrombotic event	115 (15)	6 (14)	0.890	119 (15)	2 (15)	0.999	113 (15)	8 (20)	0.407
Stroke	60 (8)	6 (14)	0.150	65 (8)	1 (7)	0.873	62 (8)	4 (10)	0.731
TIA	51 (7)	1 (2)	0.262	51 (7)	1 (7)	0.928	48 (6)	4 (10)	0.394
Peripheral embolism	9 (1)	0 (0)	1.000	9 (1)	…	1.000	9 (1)	…	1.000
β‐Blocker	271 (36)	14 (33)	0.722	277 (36)	8 (57)	0.094	270 (36)	15 (37)	0.925
Calcium channel blocker	5 (1)	1 (2)	0.280	6 (1)	…	1.000	6 (1)	0 (0)	1.000
Digoxin	1 (0)	0 (0)	1.000	1 (0)	…	1.000	1 (0)	0 (0)	1.000
ECG									
PR interval, ms	174±31	181±30	0.143	174±31	191±33	0.047	172±29	216±41	<0.001
QRS width, ms	94±20	94±20	0.938	94±20	94±10	0.957	94±20	97±19	0.455
QTc, ms	437±27	436±27	0.820	437±27	430±29	0.397	437±27	432±26	0.278
SAECG									
Filtered *P‐*wave duration, ms	141±16	148±17	0.004	141±16	144±18	0.490	141±16	145±16	0.195
Echocardiography									
LVEF, %	62±6	62±5	0.760	62±6	61±6	0.575	62±6	61±5	0.195
LVEDD, mm	47±7	46±6	0.355	47±7	50±4	0.173	47±7	50±7	0.009
LVMMI, g/m^2^	116±36	116±34	0.931	116±36	128±31	0.242	115±35	133±45	0.006
LAVI, mL/m^2^	29±10	32±9	0.120	29±10	32±10	0.363	29±9	31±14	0.235
PA‐TDI, ms	132±25	138±18	0.148	132±25	138±30	0.386	131±25	143±28	0.010
Laboratory analysis									
Creatinine, μmol/L	89±27	88±27	0.827	89±25	117±67	<0.001	89±27	95±30	0.208
hs‐CRP, mg/L (sqrt)	1.47±0.87	1.72±1.22	0.089	1.47±0.88	1.88±1.22	0.107	1.48±0.90	1.42±0.75	0.667
BNP, pg/mL (ln)	3.85±0.90	4.16±0.85	0.035	3.86±0.91	4.19±0.77	0.200	3.86±0.90	3.99±0.99	0.421
hs‐TNT, μg/L (sqrt)	0.11±0.05	0.12±0.04	0.057	0.11±0.05	0.14±0.07	0.028	0.11±0.05	0.11±0.03	0.688

Data are shown as number (percentage) or mean±SD, as appropriate. BMI indicates body mass index; BNP, brain natriuretic peptide; hs‐CRP, high‐sensitivity C‐reactive protein; hs‐TNT, high‐sensitivity troponin T; LAVI, left atrial volume index; LVEDD, left ventricular end‐diastolic diameter; LVEF, left ventricular ejection fraction; LVMMI, left ventricular myocardial mass index; PA‐TDI, total atrial conduction time as assessed by tissue Doppler imaging; QTc, corrected QT interval; SAECG, signal‐averaged ECG; SND, sinus node dysfunction; sqrt, square root; SVT, supraventricular tachycardia; and TIA, transient ischemic attack.

**Table 4:** Multivariable Analysis of Predictors of Specific Incidental Arrhythmias

Variable	Full model			Selected model		
	Coefficient (95% CI)	aOR (95% CI)	*P* value	Coefficient (95% CI)	aOR (95% CI)	*P* value
Supraventricular tachycardia						
Coronary artery disease	−0.96 (−1.83 to −0.08)	0.38 (0.16 to 0.92)	0.032	−1.02 (−1.88 to −0.16)	0.36 (0.15 to 0.85)	0.020
Filtered *P‐*wave duration, ms	0.02 (0.01 to 0.04)	1.03 (1.01 to 1.04)	0.002	0.03 (0.01 to 0.04)	1.03 (1.01 to 1.04)	0.002
hs‐CRP, mg/L (sqrt)	0.15 (−0.22 to 0.52)	1.16 (0.80 to 1.68)	0.438	…	…	…
BNP, pg/mL (ln)	0.32 (−0.03 to 0.67)	1.38 (0.97 to 1.95)	0.070	0.35 (0.00 to 0.69)	1.42 (1.00 to 2.00)	0.048
hs‐TNT, μg/L (sqrt)	3.07 (0.05 to 6.08)	21.5 (1.05 to 439.0)	0.046	3.30 (0.32 to 6.28)	27.12 (1.37 to 536.15)	0.030
Sinus node dysfunction						
Arterial hypertension	1.23 (−0.83 to 3.29)	3.43 (0.44 to 26.90)	0.240	…	…	…
Peripheral artery disease	0.76 (−0.75 to 2.28)	2.14 (0.47 to 9.73)	0.324	…	…	…
β‐Blocker	0.36 (−0.83 to 1.55)	1.43 (0.44 to 4.70)	0.555	…	…	…
PR interval, ms	0.01 (−0.00 to 0.02)	1.01 (1.00 to 1.02)	0.142	0.01 (0.00 to 0.03)	1.01 (1.00 to 1.03)	0.034
Creatinine, μmol/L	0.01 (0.00 to 0.03)	1.01 (1.00 to 1.03)	0.029	0.02 (0.01 to 0.03)	1.02 (1.01 to 1.03)	0.001
hs‐TNT, μg/L (sqrt)	1.60 (−1.72 to 4.93)	4.97 (0.18 to 138.20)	0.344	…	…	…
Atrioventricular conduction impairment						
Age, y	0.01 (−0.06 to 0.08)	1.01 (0.95 to 1.08)	0.714	…	…	…
Sex, women	−0.03 (−0.88 to 0.83)	0.97 (0.41 to 2.30)	0.968	…	…	…
Palpitations in past 12 mo	0.63 (−0.18 to 1.44)	1.87 (0.83 to 4.21)	0.130	…	…	…
PR interval, ms	0.03 (0.02 to 0.04)	1.03 (1.02 to 1.04)	<0.001	0.03 (0.02 to 0.04)	1.03 (1.02 to 1.04)	<0.001
LVEDD, mm	0.06 (−0.02 to 0.14)	1.06 (0.98 to 1.15)	0.140	…	…	…
LVMMI, g/m^2^	−0.00 (−0.01 to 0.01)	1.00 (0.99 to 1.01)	0.915	…	…	…
PA‐TDI, ms	0.00 (−0.01 to 0.02)	1.00 (0.99 to 1.02)	0.604	…	…	…

aOR indicates adjusted odds ratio; BNP, brain natriuretic peptide; hs‐CRP, high‐sensitivity C‐reactive protein; hs‐TNT, high‐sensitivity troponin T; LVEDD, left ventricular end‐diastolic diameter; LVMMI, left ventricular myocardial mass index; PA‐TDI, total atrial conduction time as assessed by tissue Doppler imaging; and sqrt, square root.

**Table 5:** Overview of Incidental Findings

Incidental findings	Total (N=794)	First 7‐d Holter ECG (N=794)		Second 7‐d Holter ECG (N=667)		Third 7‐d Holter ECG (N=616)	
		First manifestation	Overall	First manifestation	Overall	First manifestation	Overall
Atrial fibrillation	29 (3.7)	15 (1.9)	15 (1.9)	5 (0.6)	20 (2.5)	9 (1.1)	29 (3.7)
Sustained SVT	42 (5.3)	17 (2.1)	17 (2.1)	18 (2.3)	36 (4.5)	7 (0.9)	42 (5.3)
Sustained VT	2 (0.3)	2 (0.3)	2 (0.3)	…	2 (0.3)	…	2 (0.3)
Sinus node dysfunction	14 (1.8)	8 (1.0)	8 (1.0)	…	8 (1.0)	6 (0.8)	14 (1.8)
Atrioventricular conduction impairment	41 (5.2)	26 (3.3)	26 (3.3)	10 (1.3)	36 (4.5)	5 (0.6)	41 (5.2)
Second‐degree AVB	36 (4.5)	23 (2.9)	23 (2.9)	9 (1.1)	32 (4.0)	4 (0.5)	36 (4.5)
2:1 AVB	13 (1.5)	5 (0.6)	5 (0.6)	5 (0.6)	10 (1.3)	3 (0.4)	13 (1.6)
Complete AVB	8 (1.0)	6 (0.8)	6 (0.8)	1 (0.1)	7 (0.9)	1 (0.1)	8 (1.0)

Data are shown as number (percentage). AVB indicates atrioventricular block; SVT, supraventricular tachycardia; and VT, ventricular tachycardia.

### Sinus Node Dysfunction

We found SND in 14 patients (1.8%; Table 3; Figure 1). The median duration of sinus pauses was 4 seconds (interquartile range, 4–4 seconds). One patient had newly diagnosed paroxysmal AF with a sinus pause of 9 seconds after spontaneous termination of AF during the day. This patient had a pacemaker implanted (Table S1). In univariable analysis, PR interval, peripheral artery disease, creatinine, and high‐sensitivity troponin T were predictors for SND (Table 3), whereas, in the multivariable analysis, both PR interval and creatinine emerged as predictors for SND (Table 4). Table 5 shows during which Holter ECG SND manifested for the first time.

### Sustained SVT

A sustained SVT was recorded in 42 patients (5.3%; Table 3; Figure 1). Median duration of SVT was 2 minutes (interquartile range, 1–15 minutes), and the median heart rate was 145 beats per minute (interquartile range, 118–163 beats per minute). Treatment with a β‐blocker or an increased β‐blocker dose was recommended in 3 patients. One patient with symptomatic, probably typical atrioventricular nodal reentry tachycardia was offered an electrophysiological study and ablation, which he declined. In univariable analysis, filtered *P‐*wave duration and brain natriuretic peptide were significant predictors (Table 3). In multivariable analysis, both remained significant, and presence of coronary artery disease and high‐sensitivity troponin T were also predictive (Table 4). Sustained SVT mainly manifested in the first and second 7‐day Holter ECG and only rarely in the third ECG (Table 5).

### Sustained VT

In 2 patients (0.3%), we observed a sustained VT (Figure 1). One patient was an 80‐year‐old woman with a VT that lasted 38 seconds at 150 beats per minute in her first 7‐day Holter ECG. Her left ventricular ejection fraction was 50%. As a result, the β‐blocker dose was increased, and no further sustained VTs were observed during subsequent Holter ECGs. The other patient was a 69‐year‐old woman with a syncopal VT of 3 minutes’ duration at 216 beats per minute in her first 7‐day Holter ECG. She had normal left ventricular ejection fraction, but cardiac magnetic resonance imaging showed an infero‐postero‐lateral scar region. Subsequently, she was implanted with an implantable cardioverter‐defibrillator.

### Death

One patient with advanced cancer disease died during a 7‐day Holter ECG, which showed progressive bradycardia and finally asystole.

### Adverse Effects of 7‐Day Holter ECG

**Figure 2:**
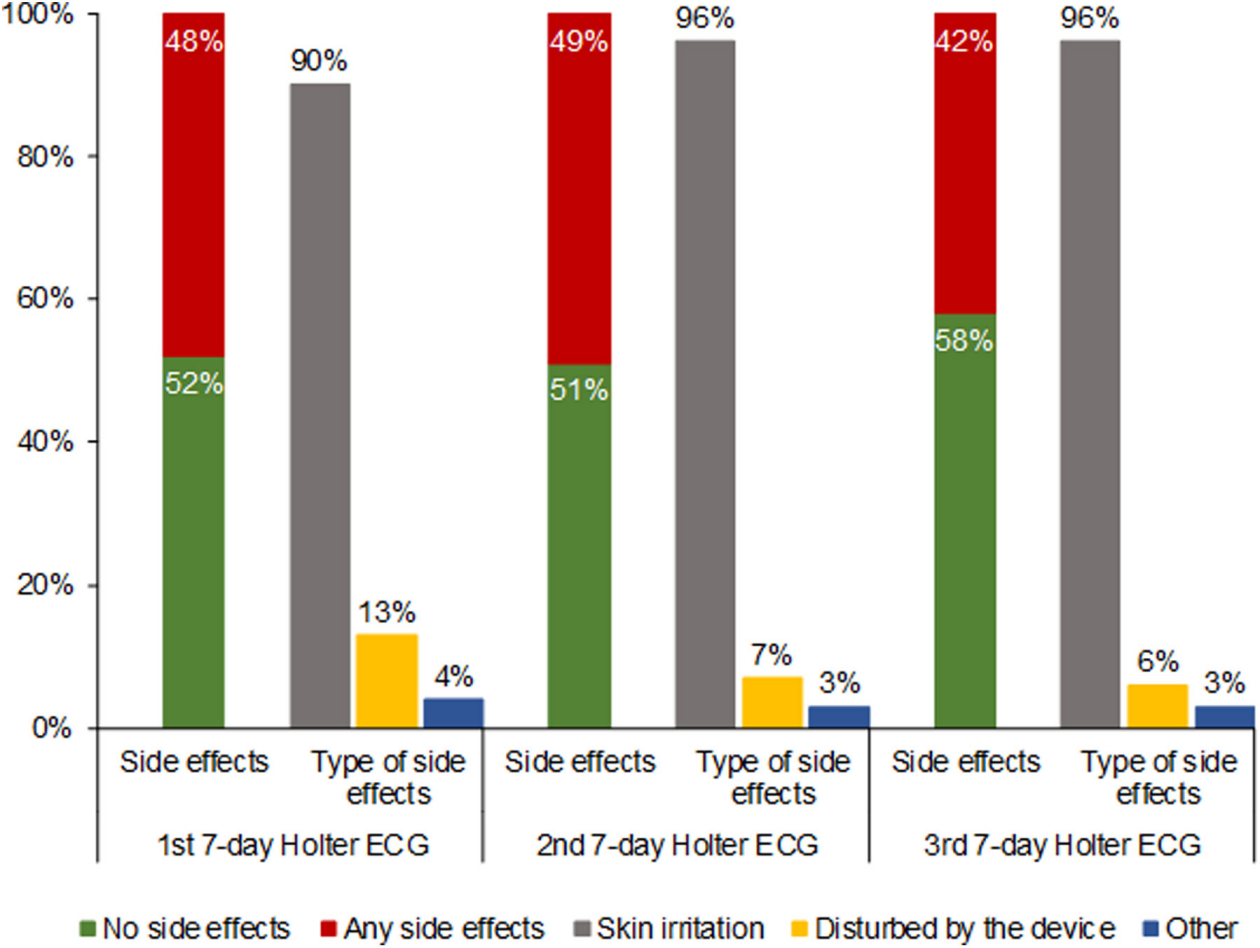
Incidence of adverse effects during Holter ECG monitoring. Bar graph showing the percentage of patients indicating adverse effects as well as specific adverse effects during the time they wore the first, second, and third 7‐day Holter ECG.

The diary that was distributed to all patients together with the 7‐day Holter ECG with questions on adverse effects was completed and sent back by 560 patients (71%) together with the first 7‐day Holter ECG, and by 520 (78%) and 498 (81%) together with the second and third 7‐day Holter ECGs, respectively. Almost half of the patients indicated adverse effects during 7‐day Holter ECGs (Figure 2). The most frequent adverse effect of the 7‐day Holter ECG was skin irritation, followed by being disturbed by the device (Figure 2). After the first 7‐day Holter ECG, 92 patients (12%) refused a further 7‐day Holter ECG, and after the second 7‐day Holter ECG, another 34 patients (5%) refused a third 7‐day Holter ECG.

## Discussion

**Figure 3:**
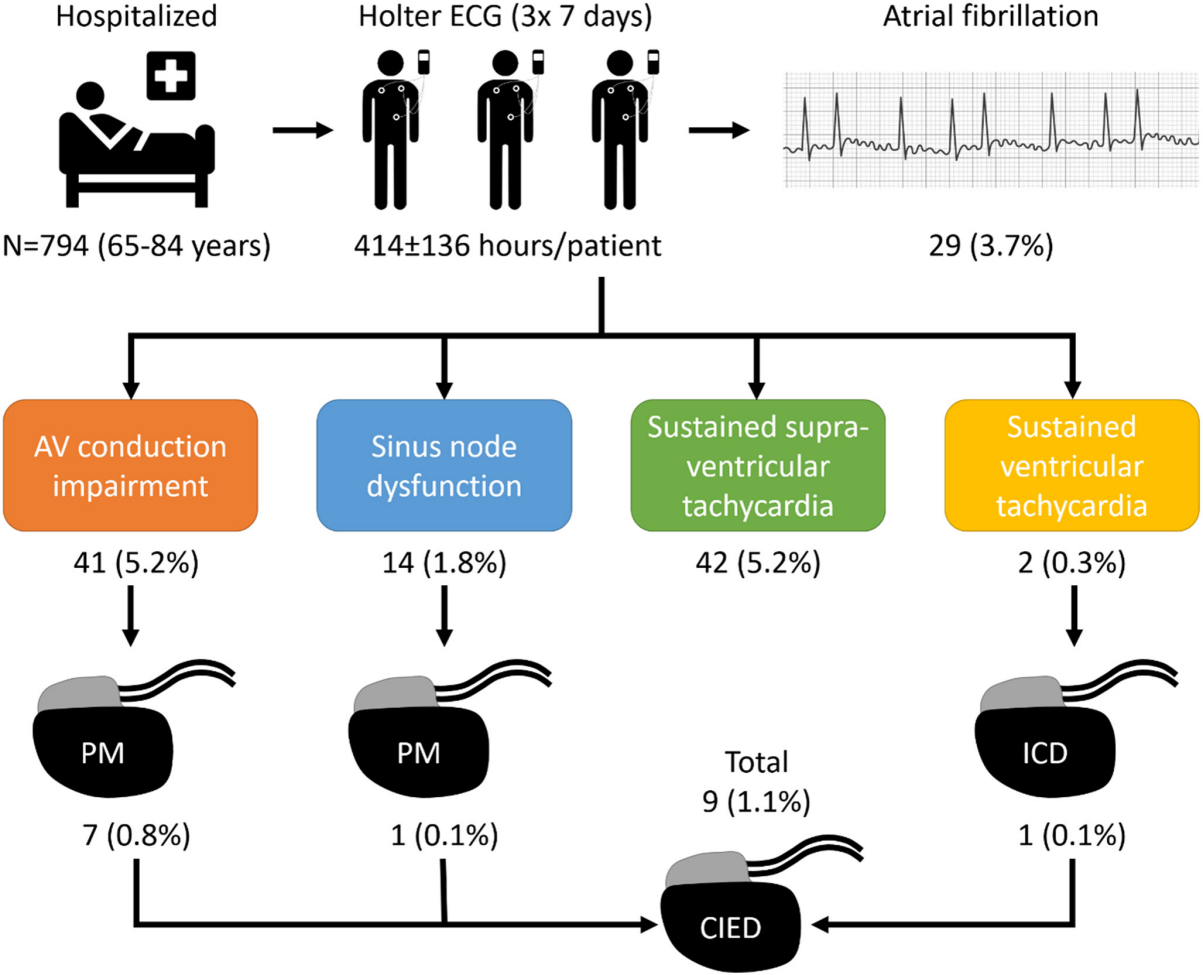
Overview of study findings. The patient population, the mean duration of Holter ECG monitoring per patient, and the incidence of incidental findings as well as consecutive device therapies are shown. AV indicates atrioventricular; CIED, cardiac, implantable, electronic device; ICD, implantable cardioverter‐defibrillator; and PM, pacemaker.

With an incidence of 12%, incidental arrhythmias were frequent during screening for AF in the STAR‐FIB cohort study. However, most of these incidental arrhythmias did not affect the treatment of the study patients. Nevertheless, 1.1% of patients finally received a cardiac implantable electronic device because of an incidental arrhythmia diagnosed during AF screening (Figure 3).

Screening for AF mainly fulfills the principles of screening defined in a landmark publication by Wilson and Jungner in 1968.^13^ However, as with other screening programs, it is important to recognize that AF screening can also lead to harm for several reasons^13^: (1) adverse effects can occur because of the screening tool; (2) both false positives as well as false negatives can result in harm; (3) detection and treatment of early stages of AF can lead to overdiagnosis and overtreatment; (4) screening can divert health resources away from other essential fields of care; and finally (5) incidental arrhythmias diagnosed during screening may lead to additional treatment that may be unnecessary and even harmful. To understand whether a screening program will have a benefit at a reasonable cost, we need to consider all potential benefits and harms. In this equation, incidental arrhythmias and their sequelae need to be included. However, most screening studies do not specifically report incidental arrhythmias of screening efforts. Only 2 AF screening studies, to our knowledge, reported on incidental arrhythmias to date: the TRACK‐AF study and the LOOP study.^14^, ^15^ In the TRACK‐AF study, implantable cardiac monitors were used to screen for AF in 173 patients with cryptogenic stroke. During a mean follow‐up of 2 years, incidental findings were reported in 8.7% of patients and were mainly short episodes of sinus pause at night. Symptomatic sinus pauses or bradyarrhythmia required pacemaker implantation in 2.9% of patients in the TRACK‐AF study. In the recently published LOOP study, bradyarrhythmias were found in 20.8% of 1501 patients implanted with a cardiac monitor.^15^ Most bradyarrhythmias were asymptomatic and, ultimately, a pacemaker was implanted in 4.5% of patients in the LOOP study. The CARISMA study was another study using implantable loop recorders to specifically screen for both bradyarrhythmias and tachyarrhythmias in patients after an acute myocardial infarction and with a left ventricular ejection fraction of ≤40%.^16^ The authors reported incidences of high‐degree AVB, sinus arrest ≥5 seconds, and sustained VT of 10%, 5%, and 3%. A pacemaker or an implantable cardioverter‐defibrillator was implanted in 4.4% of study participants.

The modality used for AF screening will affect both the incidence of an AF diagnosis as well as the incidence of other incidental arrhythmias.^17^ Because incidental arrhythmias, like SND or advanced AVB, only occur for a few seconds, the likelihood that these arrhythmias are diagnosed with only intermittent ECG monitoring is low, whereas longer‐lasting episodes of AF can be detected with reasonable sensitivity.^17^ The longer a continuous ECG is recorded, the more likely a sinus pause or a complete AVB with ≥2 nonconducted *P* waves is detected. However, diagnosis also depends on the way the ECG is analyzed. We used advanced software functionality and manual confirmatory analysis and, with this approach, we probably detected any relevant sinus pause and most of the episodes with advanced AVB. On the other hand, even with implantable cardiac monitors, short episodes of complete AVB with only a few nonconducted *P* waves may go undetected, even when the trigger for pause alerts is set to 3 seconds.

Most important, overtreatment of incidental arrhythmias should be avoided. For example, with a prevalence of 5%, atrioventricular conduction impairment was a frequent finding during a 7‐day Holter ECG in our elderly patient population. Mostly, it has an extrinsic cause with nocturnal occurrence and does not need to be treated. Therefore, in patients with advanced AVB, we only implanted a pacemaker if there was no sign of an extrinsic cause and after comprehensive evaluation and informed decision‐making, also considering the patient history.

First‐degree AVB was both predictive for more advanced AVB as well as for SND. It is not surprising that first‐degree AVB is a predictor of more advanced AVB. PR interval is a well‐described predictor for AF, and SND often occurs together with AF and shares common risk factors.^18^ A longer *P‐*wave duration suggests atrial cardiomyopathy and fibrosis and is also a known predictor for AF and, in our cohort, for sustained SVT.^19^

AF screening programs typically struggle with both patient acceptance and adherence. For example, in the mSToPS randomized clinical trial, <3% of invited patients finally participated in the screening study, and approximately one‐third of the participating patients never wore the ECG patch.^4^ Similarly, in the AHS (Apple Heart Study), only one‐fifth of participants with irregular pulse notifications returned the ECG patch with data that could be analyzed.^3^ In the STROKESTOP I Study and the REHEARSE‐AF Study, only 50% and 20% of invited subjects participated, respectively. However, both studies used intermittent, simple handheld ECG recordings to screen for AF, which are convenient to use for the patients.^2^, ^6^ With continuous 7‐day Holter ECG, half of our patients reported adverse effects, mainly skin irritation. This issue is always present with continuous ECG recording using ECG patches or electrodes. Some patients were also troubled by the device and its cables, which can be avoided using ECG patches instead of classic Holter devices. Nevertheless, ≈80% of study participants completed the study according to protocol with 3 repeat 7‐day Holter ECGs. More important, our patients received spare electrodes and instructions to change electrode positions daily, to avoid skin irritation, and patients were allowed to take the device off (eg, for showering), which all may have helped to increase patient adherence.

### Limitations

The results of our study only apply to a hospitalized patient population and cannot be generalized to the general population. The findings also depend on the tools used for AF screening: other screening tools may find a different incidence of incidental arrhythmias. The clinical relevance of incidental arrhythmias and treatment decisions may be debatable.

## Conclusions

Incidental arrhythmias were frequent during screening for AF in the STAR‐FIB study and resulted in device therapy in 1.1% of our cohort patients, mainly because of advanced AVB.

## Sources of Funding

This work was supported by a grant of the Swiss National Science Foundation (grant number 32003B_156292); and by the Swiss Heart Foundation.

## Disclosures

Dr Neugebauer has received travel/educational grants from Medtronic, Abbott, Boston Scientific, and Philips/Spectranetics. Dr Kozhuharov has received research grants from the Swiss National Science Foundation (P400PM‐194 477 and P5R5PM_210856), Gottfried und Julia Bangerter‐Rhyner‐Stiftung, Freiwillige Akademische Gesellschaft, L. & Th. La Roche Stiftung, and the European Society of Cardiology. Dr Haeberlin has received travel fees/educational grants from Medtronic, Philips/Spectranetics, and Biotronik without impact on his personal remuneration. He serves as a proctor for Medtronic. He has received research grants from the Swiss National Science Foundation, the Swiss Heart Foundation, the University of Bern, the University Hospital Bern, the Velux Foundation, the Hasler Foundation, the Swiss Heart Rhythm Foundation, and the Novartis Research Foundation. He is cofounder and CEO of Act‐Inno, a cardiovascular device testing company. Dr Reichlin has received research grants from the Goldschmidt‐Jacobson Foundation, the Swiss National Science Foundation, the Swiss Heart Foundation, and the sitem insel support fund, all for work outside the submitted study. He has received speaker/consulting honoraria or travel support from Abbott/SJM, Bayer, Biosense‐Webster, Biotronik, Boston‐Scientific, Daiichi Sankyo, Farapulse, Medtronic, and Pfizer‐BMS, all for work outside the submitted study. He has received support for his institution's fellowship program from Abbott/SJM, Biosense‐Webster, Biotronik, Boston‐Scientific, and Medtronic for work outside the submitted study. Dr Roten has received speaker/consulting honoraria from Abbott/SJM and from Medtronic and has received a research grant to the institution from Medtronic. The remaining authors have no conflicts to disclose.

## Supporting information

Table S1.
