# Supplementary material for: Incidental Arrhythmias During Atrial Fibrillation Screening With Repeat 7‐Day Holter ECGs in a Hospital‐Based Patient Population
Source: J Am Heart Assoc. 2024 Feb 13;13(4):e032223. doi: 10.1161/JAHA.123.032223 (PMC11010089; doi:10.1161/JAHA.123.032223)
Supplement: Supplementary file 1 — Table S1. [file JAH3-13-e032223-s001.pdf]

SUPPLEMENTAL MATERIAL

Table S1. Patient characteristics and findings in 7-day Holter ECG of patients that received a cardiac implantable electronic device.

Case#	Holter	Age	Sex	Finding	Pause, sec.	Description of incidental arrhythmias	Device
156	3rd	65y	Male	AVB	3	QRS narrow, in the evening, no signs of extrinsic aetiology	Leadless PM
229	1st	79y	Female	AVB	6	QRS narrow, during the day, no signs of extrinsic aetiology	DDD-PM
252	1st	75y	Male	AVB	3	QRS narrow, during the day, no signs of extrinsic aetiology, history of syncope	DDD-PM
257	1st	76y	Male	AVB	18	QRS narrow, during night, history of syncope	DDD-PM
419	2nd	84y	Male	AVB	-	2:1 AVB, complete AVB with junctional escape rhythm, history of syncope	DDD-PM
663	1st	65y	Female	AVB	-	2:1 AVB, complete AVB with junctional escape rhythm during the day	DDD-PM
724	1st	68	Female	VT	-	VT (216 bpm; 3 minutes) with syncope, LV scar, normal LVEF	VVI-ICD
778	1st	84y	Female	AVB	3	2:1 AVB, QRS narrow, during the day, history of syncope	DDD-PM
803	3rd	73y	Female	SND	9	AF with pause after spontaneous cardioversion	DDD-PM

AVB: atrioventricular block; AF: atrial fibrillation; ICD: implantable cardioverter-defibrillator; LV: left ventricular; LVEF: left ventricular ejection fraction; PM: pacemaker; VT: ventricular tachycardia.
